# Mediators of Effects on Physical Activity and Sedentary Time in an Activity Tracker and Behavior Change Intervention for Adolescents: Secondary Analysis of a Cluster Randomized Controlled Trial

**DOI:** 10.2196/35261

**Published:** 2022-08-16

**Authors:** Simone Johanna Josefa Maria Verswijveren, Gavin Abbott, Samuel K Lai, Jo Salmon, Anna Timperio, Helen Brown, Susie Macfarlane, Nicola D Ridgers

**Affiliations:** 1 Institute for Physical Activity and Nutrition School of Exercise and Nutrition Sciences Deakin University Burwood Australia; 2 Learning Futures Deakin University Geelong Australia; 3 Alliance for Research in Exercise, Nutrition and Activity University of South Australia Adelaide Australia

**Keywords:** movement behavior, youth, accelerometry, Fitbit, correlates, correlate, physical activity, exercise, randomized controlled trial, RCT, control trial, Australia, adolescent, adolescence, teenager, sedentary, cognitive theory, behavioral theory, wearable, tracker, tracking device, clinical trial

## Abstract

**Background:**

Adolescence is a critical age where steep declines in physical activity and increases in sedentary time occur. Promoting physical activity should therefore be a priority for short- and long-term health benefits. Wearable activity trackers in combination with supportive resources have the potential to influence adolescents’ physical activity levels and sedentary behavior. Examining the pathways through which such interventions work can inform which mediators to target in future studies.

**Objective:**

The aim of this paper is to examine the impact of the Raising Awareness of Physical Activity (RAW-PA) intervention on potential mediators of behavior change after intervention, and whether these mediated the intervention effects on physical activity and sedentary time at 6-month follow-up.

**Methods:**

RAW-PA was a 12-week intervention, grounded in social cognitive theory and behavioral choice theory, aimed at increasing physical activity among inactive adolescents through combining a wearable activity tracker with digital resources delivered via a private Facebook group (n=159 complete cases). The targeted potential mediators were identified from previous studies conducted in adolescents and included self-efficacy, peer support, family support, teacher support, self-regulation strategies, barriers, and enjoyment. Outcomes included sedentary time as well as light- and moderate-to-vigorous–intensity physical activity. A series of mixed linear models were used to estimate intervention effects on physical activity and sedentary behavior at follow-up and on potential mediators after intervention and to test whether there were indirect effects of the intervention on physical activity and sedentary behavior via mediators.

**Results:**

Adolescents in the intervention group (n=75) engaged in higher sedentary time and lower light intensity at 6-month follow-up compared to the wait-list controls (n=84). There were no intervention effects for moderate-to-vigorous–intensity physical activity. The intervention group perceived more barriers to physical activity than the wait-list control group at 6-month follow-up (mean adjusted difference=1.77; 95% CI 0.19-3.34; *P*=.03). However, indirect effects for each outcome were not statistically significant, indicating that perceived barriers to physical activity did not mediate intervention effects for physical activity or sedentary time.

**Conclusions:**

RAW-PA did not beneficially impact hypothesized mediators in these inactive adolescents, despite strategies being designed to target them. This suggests that the lack of overall intervention effects on physical activity and sedentary time observed in the RAW-PA study could be due to the limited impact of the intervention on the targeted mediators. Future studies should consider different strategies to target theoretically informed potential mediators and identify intervention strategies that effectively target key mediators to improve physical activity among inactive adolescents. Finally, intervention effects according to level of wearable tracker use or level of engagement with the intervention should be explored. This may provide important insights for designing successful wearable activity tracker interventions.

**Trial Registration:**

Australian New Zealand Clinical Trials Registry ACTRN12616000899448; https://anzctr.org.au/Trial/Registration/TrialReview.aspx?id=370716&isReview=true

**International Registered Report Identifier (IRRID):**

RR2-10.1186/s12889-016-3945-5

## Introduction

Most adolescents worldwide do not engage in sufficient physical activity to benefit health, including cardiometabolic health, and cognitive development [1,2]. This high prevalence of inactivity is evident across low-, middle-, and high-income countries [1]. By contrast, emerging research has shown that excessive sedentary behavior, which includes activities such as TV viewing, may be detrimentally associated with adolescents’ health [3]. As adolescence is a critical age where steep declines in physical activity occur [4], particularly in adolescents living in areas of socioeconomic disadvantage [5], and as youth engage more in total sedentary time when they grow older [6], promoting physical activity should be a priority for short- and long-term health benefits [7].

The past decade has seen advances in the development of commercially available wearable activity trackers. Such devices can measure physical activity, enable self-monitoring, and provide real-time feedback. Wearable activity trackers are increasingly being used in physical activity research as both a measurement and intervention tool, while some devices have the capability to provide feedback and alerts for periods of sedentary time [8,9]. Emerging evidence suggests that these trackers have the potential to be used as intervention tools as adolescents perceive them as easy to use and useful [10], and that they increase their motivation to be physically active [11]. However, surprisingly few studies have used activity trackers in physical activity interventions for adolescents, and the scarce evidence is mixed. While one review showed that these devices are feasible for increasing physical activity and decreasing sedentary behavior [12], others have found that effectiveness for physical activity, particularly long-term, has not yet been established [9,11]. More research is required to understand the effect of using activity trackers on adolescents’ daily behaviors, which include physical activity of different intensities and sedentary time [13].

Previous technology-based physical activity interventions using activity trackers have shown that multicomponent interventions may be more effective than using wearables as a stand-alone strategy [9,11]. That is, combining activity trackers with additional resources may be a useful strategy to change physical activity behaviors [14]. Examples include web-based advice to increase social support [14,15], tailored advice [16], incentives [17], and counselling [18] in combination with activity trackers. Although some recent activity tracker interventions have started to incorporate additional components [9,12], no previous activity tracker studies have examined potential mediators of change in physical activity and sedentary behavior. Such evidence would provide insights into why the interventions may or may not have been effective. Examining mediators of behavior change may help to identify the pathway through which activity tracker interventions work in adolescents [19,20] and may inform the design and delivery of such interventions in the future.

The Raising Awareness of Physical Activity (RAW-PA) study was a multicomponent intervention for adolescents living in areas of socioeconomic disadvantage that aimed to integrate more physical activity into their day by combining an activity tracker with digital resources that specifically targeted evidence-based behavior-change techniques. Grounded in social cognitive theory [21] and behavioral choice theory [13], the intervention targeted multiple potential mediators of adolescent physical activity. While this intervention did not increase inactive adolescents’ accelerometer-derived and self-reported physical activity levels immediately after intervention [8], it provides a unique opportunity to explore potential cognitive, behavioral, and interpersonal mediators of physical activity and sedentary behavior. This is important as identifying which factors are on the mediating pathway between the intervention and the targeted outcomes can inform the development of future physical activity interventions. Therefore, the aim of this study was to examine the impact of the Raising Awareness of Physical Activity (RAW-PA) intervention on potential mediators of behavior change after intervention, and whether these mediated the intervention effects on physical activity and sedentary time at 6-month follow-up.

## Methods

### Ethics Approval

Ethical approval was obtained from the Deakin University Human Research Ethics Committee (2016–179) and the Victorian Department of Education and Training.

### Study Design, Recruitment, and Participants

The RAW-PA study was evaluated using a cluster-randomized controlled trial design, with schools being the unit of randomization [14]. The trial is registered with the Australian and New Zealand Clinical Trials Registry (ACTRN12616000899448). The protocol for this trial has been previously published [14]. In brief, RAW-PA combined a Fitbit Flex (wearable activity tracker; core component) and the accompanying app with digital behavior change resources that were accessible via social media. Schools were eligible to participate if they were located in socioeconomically disadvantaged suburbs based on a Socio-Economic Index for Areas (SEIFA) [22] score of ≤5. In total, 18 schools were recruited and assigned to the intervention group (n=9) or wait-list control (n=9) using a computer-based random number generator.

**Figure 1:**
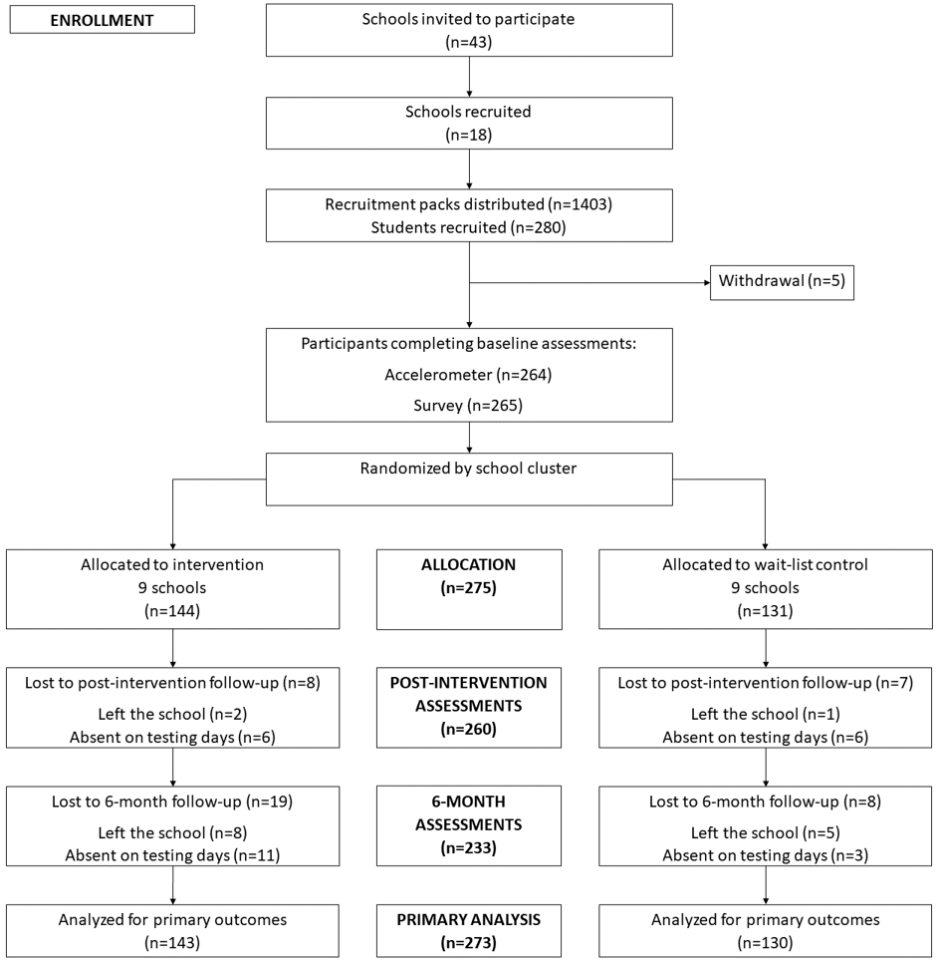
Participant flow diagram.

The eligibility criteria for participants included the following: (1) being ≥13 years old; (2) having access to the internet outside of school; (3) not engaging in regular organized physical activity or sport outside of school; (4) not meeting physical activity guidelines of ≥60 minutes of moderate to vigorous physical activity (MVPA) every day; and (5) not a current or past owner of an activity tracker. All eligible students (n=280) who returned informed written parental consent and student assent were recruited into the study [8]. As 5 students withdrew before baseline data collection, a total of 275 students participated. Of those, 2 participants did not provide any data; thus, 273 participants were considered for analyses (depending on whether they provided complete data) [8]. A flow diagram of the participants is shown in Figure 1.

### Intervention

Developed using participatory research principles [14], the 12-week intervention combined Fitbit Flex and the accompanying app with interactive individual or weekly *missions*, including behavior change resources (eg, infographics, videos, and social forums) that were accessible via a private, researcher-moderated Facebook group, and new content alerts in the student’s own time [14]. Each weekly mission focused on how to integrate low-cost, everyday physical activity into daily life to facilitate real-world implementation [23] and provided participants with opportunities to learn and practice behavior change techniques. The weekly *missions* centered on different intervention objectives (eg, social support for physical activity), and the digital behavior change resources targeted a range of behavior change techniques and mediators [14]. The behavior change techniques were mapped against key determinants or potential mediators identified from social cognitive theory [21] and behavioral choice theory [13]. These recognize that physical activity and sedentary behavior are influenced by factors operating at multiple levels including intrapersonal (eg, self-efficacy) and interpersonal (eg, peer support and influences) [14]. Seven potential mediators of physical activity and sedentary behavior were targeted, which were as follows: self-efficacy, peer support, family support, teacher support, self-regulation strategies, perceived barriers to physical activity, and physical activity enjoyment [14,19,20,24,25]. These were selected as they have been previously identified as potential mediators of physical activity (perceived barriers to physical activity, teacher support, and physical activity enjoyment) [14,19,20,24] or both physical activity and sedentary behavior (self-efficacy, peer support, family support, and self-regulation strategies) [25]. The resources matched the weekly missions to reinforce key messages. For example, in “Week 3,” social support was targeted through encouraging participants to support their peers to increase their activity levels. The intervention also incorporated 1 week of content on breaking up sitting time with physical activity. The participants in the wait-list control group were provided access to all resources after the 6-month follow-up assessments.

### Measures

All assessments were conducted at baseline (T0), immediately after intervention (T1), and at 6-month follow-up (T2) by trained research assistants using standardized protocols.

#### Accelerometry

Physical activity and sedentary time were measured using GT3X+ ActiGraph accelerometers (ActiGraph, Pensacola, Florida, USA). The participants were instructed to wear the accelerometer on the hip for 8 consecutive days at each time point during waking hours (except during water-based activities). Raw acceleration data were downloaded and processed into 15-second epochs using manufacturer software (ActiLife). Data were then processed in a customized Microsoft Excel macro to identify sedentary time and time in light-intensity physical activity (LPA) and MVPA. Nonwear time was defined as 60 minutes of consecutive zeroes [26]. Sedentary time was calculated as the total time spent in ≤25 counts per epoch [27]. Age-specific thresholds were used to determine time spent in MVPA [28]. The time spent in between the thresholds for sedentary time and MVPA was classified as LPA. Total sedentary time, LPA, and MVPA were averaged across valid days. A valid day was defined as ≥8 hours on weekdays and ≥7 hours on weekends [29]. Adolescents who provided valid data on any 3 or more days were included for analysis [29].

#### Demographic Characteristics

The participants self-reported sex, age, and date of birth at each time point via a web-based survey. Area-level socioeconomic status (SES) of the school location using the participants’ postcode was obtained via SEIFA [22]. The SEIFA scores were categorized into decile data for use in the analyses.

#### Mediators

The 7 potential mediators (self-efficacy, peer support, family support, teacher support, self-regulation strategies, perceived barriers to physical activity, and physical activity enjoyment [14,19,20,24,25]) were assessed at all time points using items that were validated for youth [30-32]. The scales used to assess self-efficacy, peer support, family support, teacher support, and self-regulation strategies were adapted from a previously designed instrument for assessing social cognitive measures related to physical activity behaviors [30]. This instrument was developed based on constructs from social cognitive theory by Bandura [21]. The scales have acceptable reliability in adolescents (Cronbach α=.69-.79; test-retest intraclass correlation=0.86-0.91) [30]. The self-efficacy scale included five items (eg, “I do not feel comfortable using local facilities to be active” [30]), which were assessed using a 6-point Likert-type scale ranging from “strongly disagree” (1) to “strongly agree” (6). Social support from peers and family was assessed using 9 items (eg, “In the past three months, how often did your friends encourage you to be active?” [30]) on a 5-point Likert-type scale ranging from “never” (1) to “always” (5). These items were then adapted to assess social support from teachers (4 items). Self-regulation strategies were assessed using 6 items with a 5-point Likert-type scale, from “never” (1) to “always” (5). Example items included “In the past three months, how often did you keep track of how much physical activity you did?” [30].

Perceived barriers to physical activity were assessed using 9 items drawn from the Adolescent Physical Activity Perceived Barriers and Benefits Scale (eg, “I have minor aches and pains from activity”) [32]. The participants responded to each item using a 4-point Likert-type scale ranging from “not at all true” (1) to “very true” (4). This scale has acceptable test-retest reliability (r=0.71) and internal consistency (Cronbach α=.79) [32] in adolescents. Enjoyment of physical activity was assessed using the 16-item Physical Activity Enjoyment Scale [31]. The participants used a 5-point Likert-type scale ranging from “Disagree a lot” (1) to “Agree a lot” (5) to answer questions such as “When I am active, I enjoy it”. The Physical Activity Enjoyment Scale has been validated for use in adolescents [31].

The overall scores for each of these variables were created by summing individual item scores. These scores were then used as indicators for self-efficacy (range: 5-30), peer support (range: 5-25), family support (range: 4-20), teacher support (range: 4-20), self-regulation strategies (range: 6-30), perceived barriers to physical activity (range: 9-36), and physical activity enjoyment (range: 16-80).

### Analytical Sample

Of the 273 participants who were considered for inclusion in the analyses, baseline accelerometer data were available for 264 (96.7%) students and survey assessments for 265 (97.1%). Valid accelerometer data were available for 246 (90.1%) participants at baseline (T0), 198 (72.5%) after intervention (T1), and 193 (70.7%) at 6-month follow-up (T2). The complete case analysis sample (n=159; 58.2%) included those with full data for covariates (sex and SES), mediators at baseline (T0) and after intervention (T1), and valid accelerometry at baseline (T0) and 6-month follow-up (T2).

### Statistical Analysis

Baseline (T0) demographic characteristics as well as baseline (T0) and 6-month follow-up (T2) accelerometry variables (mean [SD]) of the analytic sample were calculated and descriptively compared with excluded participants. As per the Consolidated Standards of Reporting Trials recommendations [33], baseline characteristics for the wait-list control and intervention groups were presented separately but were not compared using inferential tests. Inspection of histograms of the physical activity and sedentary time variables indicated all were normally distributed. Moreover, the median, 25th, and 75th percentile values were quite consistent with values expected for a normal distribution (ie, mean and mean [SD 0.67], respectively). For the mediation analyses, linear mixed models were used, with random intercepts for schools to account for clustering by schools. All models adjusted for sex, school-area SES, and average accelerometer wear time. The covariates included in this study were selected due to potential sex differences and socioeconomic differences in the adolescents’ physical activity and sedentary behavior. Wear time was also included due to differences in accelerometer wear time by adolescents in the study. Stata SE 15 (StataCorp) was used to conduct analyses, and statistical significance was set at *P*<.05.

**Figure 2:**
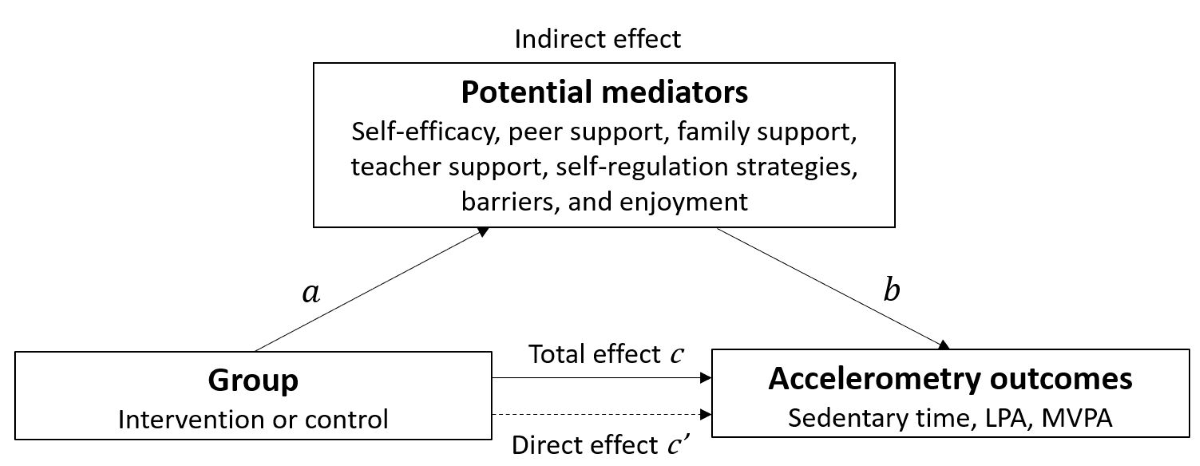
Visual representation of the hypothesized mediator model. Pathway a represents the intervention effect on potential mediators, b is the effect of the potential mediator on the behavioural outcome while adjusting for (independent of) intervention group, c is the total effect of the intervention on the outcome, c' (direct effect) is the effect of the intervention on the behavior outcome independent of the potential mediator, while the indirect effect is calculated as a×b and represents the portion of intervention effect on the outcome, which occurs via the mediator. LPA: light-intensity physical activity; MVPA: moderate-to-vigorous–intensity physical activity.

Mediation analysis of randomized controlled trials is important both when an intervention effect on the outcome is observed and when it is not [34]. In the latter case, which is relevant to this intervention [8], the inspection of intervention effects on potential mediators can help to elucidate why the intervention did not affect the outcome (ie, some mediators may play a *suppression* role), for example, because the intervention failed to impact important intermediate factors (*a* path) or that the intermediate factors did not impact the outcome as hypothesized (*b* path). Accordingly, modern approaches to mediation emphasize the importance of testing the *a* and *b* paths irrespective of the total intervention effect on the outcome. A visual representation of the hypothesized mediator model, including the several pathways assessed, is depicted in Figure 2. In this framework, the total effect is the overall effect of the exposure (intervention) on physical activity and sedentary behavior (outcome variables), while the direct effect is the effect of the exposure (intervention) on physical activity and sedentary behavior that operates independently of the mediator in question [35]. First, the models were fitted to estimate the intervention effect on each potential mediator after intervention (T1; *a* path), while adjusting for baseline levels of the mediator. For potential mediators on which there was a statistically significant intervention effect at T1, single-mediator analyses were conducted to determine the indirect effect of the intervention on physical activity and sedentary time at 6-month follow-up (T2) via the mediator (assessed after intervention [T1]). This involved fitting a model while simultaneously estimating the effects of the intervention (direct effect; *c’* path) and the potential mediator (*b* path) on each outcome variables at 6-month follow-up (T2). The mediated, or indirect, effect (ie, the portion of the exposure-outcome relationship that occurs via the mediator) was calculated as *a* × *b,* following the “product of coefficients” method, and this quantity was bootstrapped with 1000 resamples to produce percentile-based 95% CIs [35]. The models were also fitted to estimate the intervention effect on each outcome (*c* path). All models in this stage were adjusted for baseline levels of both the mediator and total physical activity or sedentary time (depending on outcome of interest).

## Results

### Participants

Baseline (T0) participant characteristics and accelerometry variables at baseline and 6-month follow-up (T2) are shown in Table 1. The participants were approximately 14 years old at baseline, and there were slightly more females than males in both groups (wait-list control: 38% [32/84]; intervention: 45% [34/75]). On average, the participants spent approximately 70% of accelerometer wear time at baseline being sedentary, 25% in LPA and 5% in MVPA. The participants in the analytic sample (n=159) were more likely to be female (93/159, 58% vs 48/114, 42%) and come from the highest eligible school SES decile (47/159, 30% vs 12/114, 11%) compared to the participants who were excluded from the analyses. However, the participants were similar in terms of baseline mediators, physical activity, and sedentary time (data not shown).

**Table 1:** Baseline (T0) demographic characteristics as well as baseline (T0) and 6-month follow-up (T2) accelerometry variables (n=159).

Characteristics				Control (n=84)		Intervention (n=75)
Baseline age (years), mean (SD)				13.7 (0.4)		13.7 (0.4)
**Sex, n (%)**						
	Male		32 (38)		34 (45)	
	Female		52 (62)		41 (55)	
**School-area SES^a^ decile, n (%)**						
	1		21 (25)		22 (29)	
	2		0 (0)		25 (33)	
	3		10 (12)		17 (23)	
	4		12 (14)		5 (7)	
	5		41 (49)		6 (8)	
**Activity variables, mean (SD)**						
	**Baseline (T0)**					
		Average daily wear time (min)	744.6 (99.0)		753.9 (149.2)	
		Average daily sedentary time (min)	512.3 (90.9)		541.3 (146.6)	
		Average daily LPA^b^ (min)	192.3 (53.6)		178.2 (40.8)	
		Average daily MVPA^c^ (min)	39.9 (18.8)		34.5 (21.0)	
	**6-month follow-up (T2)**					
		Average daily wear time (min)	769.1 (105.9)		751.3 (155.1)	
		Average daily sedentary time (min)	538.7 (100.1)		558.7 (149.6)	
		Average daily LPA (min)	193.8 (53.4)		163.5 (36.2)	
		Average daily MVPA (min)	36.6 (18.6)		29.0 (14.5)	

^a^SES: socioeconomic status.

^b^LPA: light-intensity physical activity.

^c^MVPA: moderate-to-vigorous–intensity physical activity.

### Mediation Analysis

The mean scores for each mediator at baseline (T0) and after intervention (T1) are shown in Table 2, along with estimated intervention effects after intervention (*a* path). There was evidence of an intervention effect on the perceived barriers to physical activity score after intervention, with adolescents in the intervention group perceiving more barriers to physical activity compared to those in the wait-list control group (mean adjusted difference=1.77; 95% CI 0.19, 3.34; *P*=.03). There was little evidence of intervention effects for the remaining potential mediators.

**Table 2:** Estimated effects of the intervention on potential mediators immediately after intervention (a path; n=159). Models were adjusted for sex, school socioeconomic status, accelerometer wear time, and baseline levels of potential mediators.

Mediators	Baseline (T0)			After intervention (T1)			After intervention MAD^a^	
	Control, mean (SD)	Intervention, mean (SD)	Control, mean (SD)		Intervention, mean (SD)	β (95% CI)		*P* value
Self-efficacy	19.79 (4.25)	18.60 (5.01)	19.87 (4.79)		19.29 (4.71)	0.21 (–1.12, 1.53)		.76
Peer support	15.73 (4.10)	14.83 (4.01)	16.30 (4.26)		15.40 (4.54)	0.00 (–1.44, 1.43)		.99
Family support	12.90 (3.61)	11.85 (3.88)	12.82 (3.98)		11.28 (4.28)	–0.93 (–2.14, 0.29)		.14
Teacher support	11.87 (3.49)	10.71 (3.73)	12.40 (3.82)		11.33 (4.01)	–0.45 (–1.70, 0.80)		.48
Self-regulation strategies	17.02 (3.88)	16.37 (4.71)	18.49 (4.88)		18.56 (4.72)	1.00 (–0.62, 2.61)		.23
Perceived barriers to physical activity	20.39 (4.45)	20.32 (4.59)	18.79 (5.35)		20.19 (4.88)	1.77 (0.19, 3.34)		.03
Physical activity enjoyment	64.42 (10.09)	60.93 (10.43)	63.86 (10.88)		62.03 (10.54)	–0.68 (–3.79, 2.43)		.67

^a^MAD: mean adjusted difference, where values above 0 indicate higher estimated means for the intervention group.

As the perceived barriers to physical activity were the only potential mediators for which there was a statistically significant intervention effect, formal mediation analyses were only conducted for this factor. Table 3 shows the estimated total (*c* path) and direct (*c’* path; while adjusting for the perceived barriers to physical activity score) effects of the intervention on physical activity and sedentary behavior at 6-month follow-up (T2; *c’* path). The total effects indicated that adolescents in the intervention group engaged in higher sedentary time and lower LPA at 6-month follow-up (T2) compared to the wait-list controls. These differences were still observed while adjusting for the perceived barriers to physical activity score (ie, direct effect).

Table 3 also presents the estimated indirect effects, defined as the effect of the intervention on physical activity and sedentary time at 6-month follow-up (T2) that occurred via the perceived barriers to physical activity score after intervention (T1). However, the indirect effects for each outcome were not statistically significant, indicating that the perceived barriers to physical activity did not mediate the intervention effects for physical activity or sedentary time.

**Table 3:** Estimated total (c path), direct (c' path), and indirect (a × b) effects of the intervention on 6-month follow-up (T2) physical activity and sedentary behavior, and indirect effects of the intervention on 6-month follow-up (T2) physical activity and sedentary behavior via the perceived barriers to physical activity mediator immediately after intervention (T1; n=159).

Activity variables	Total effect^a^		Direct effect			Indirect effect	
	*c* path (95% CI)	*P* value^b^	*c’* path (95% CI)	*P* value	*a* × *b* (95% CI)		*P* value^b^
Sedentary time	21.23 (3.57, 38.89)	.02	22.52 (4.76, 40.28)	.01	–1.28 (–4.93, 1.16)		>.05
Light-intensity physical activity	–15.87 (–27.86, –3.89)	<.01	–16.41 (–28.57, –4.24)	<.01	0.54 (–1.45, 3.49)		>.05
Moderate-to-vigorous–intensity physical activity	–6.33 (–13.97, 1.31)	.10	–6.74 (14.28, 0.80)	.08	0.48 (–0.16, 1.83)		>.05

^a^Direct and indirect effects may not exactly add up to the total effect due to variations in school-level effects between models.

^b^Exact *P* values not readily available for asymmetric percentile-based confidence intervals.

## Discussion

### Principal Results

This study aimed to examine the impact of the RAW-PA intervention on potential mediators of behavior change after intervention, and whether these mediated the intervention effects on physical activity and sedentary time at 6-month follow-up. While the intervention had a significant adverse effect on the perceived barriers to physical activity after intervention (*a* path), with adolescents in the intervention group perceiving more barriers than those in the wait-list control group, this did not mediate the intervention effects on physical activity or sedentary time at 6-month follow-up. No intervention effects were observed on any of the remaining factors identified as potential mediators, despite the intervention aiming to change target variables (eg, self-efficacy) hypothesized to be causally related to youth physical activity and sedentary time [14,20].

While there has been some variability of intervention effects on the barriers assessed in previous studies conducted in youth [20,36,37], the intervention effects on the barriers to physical activity in this study are consistent with several studies that have reported an increase in perceived barriers following the intervention [38-40]. Within RAW-PA, adolescents were asked to reflect on the potential barriers to engaging in physical activity, and a range of strategies for overcoming barriers and integrating activity into daily life were targeted via the weekly missions and the accompanying behavior change resources [14]. It is possible, however, that this may have increased the adolescents’ awareness of potential barriers, or they may have encountered barriers more frequently when attempting to increase their activity levels [39]. It is also possible that the intervention dose may not have been sufficient to overcome these barriers. Alternatively, the activity tracker itself may have affected perceived barriers. A key component of this technology is the ability to self-monitor physical activity, which has been shown to be an effective behavior change technique and critical for changing behavior [41]. However, this may have increased the adolescents’ awareness of low activity levels, thus reinforcing barriers to being active.

While the analyses found evidence of an intervention effect on the perceived barriers to physical activity, there was no evidence of an indirect mediating effect. That is, the perceived barriers to physical activity after intervention did not mediate physical activity and sedentary time at 6-month follow-up. This suggests that the change in perceived barriers may not be on the causal pathway [42], which is consistent with the findings of several previous studies conducted in adolescents [39,40], albeit using different intervention strategies. The total effects observed may be explained by variables that were not assessed within this intervention, such as those that may be specific to the use of the activity tracking technology (eg, goal focus) [43], which was a core component within RAW-PA. Future research projects using activity trackers should consider examining a broader range of potential mechanisms drawn from different theories (eg, the technology acceptance model [44]) that focus on how adolescents perceive and use such devices.

To date, few activity tracker interventions, regardless of population group, have examined mediators of physical activity and sedentary time. A recent review highlighted that activity tracker studies have instead focused on mediators of outcomes such as task motivation in adult populations, with intervention effects observed for mediators including self-efficacy and self-awareness [43]. Despite this, the findings from this study are consistent with those from previous physical activity interventions conducted with adolescents who have reported few or inconsistent intervention effects on assessed mediators [15,20,25]. This suggests that the combination of activity trackers and digital resources may not beneficially change the targeted mediators or that that the targeted mediators were not the most effective for changing physical activity and sedentary behavior among inactive adolescents. It may be that other mediators of physical activity (eg, emotion [41]) should be targeted. Nevertheless, this supports previous research [15] that also showed few intervention effects on the determinants of activity but did not use mediation analysis. However, it should be noted that the RAW-PA implementation evaluation found that engagement with digital resources (eg, social media posts and challenges) was low across the intervention, with only 36% of adolescents in the intervention group reporting having completed the weekly challenges [23]. This may have contributed to the lack of effects observed, as the resources related to key behavior change techniques may not have been accessed and therefore utilized by adolescents in the intervention group. This study did not collect data concerning potential mediators of activity tracker use specifically, and future studies may investigate this by exploring intervention effects according to the level of wearable tracker use or the level of engagement with the intervention. This will provide greater insights into what elements worked best and what to target in future strategies.

### Strengths and Limitations

This is the first study, to the authors’ knowledge, to examine potential mediators of change in physical activity and sedentary time during an activity tracker intervention conducted in adolescents. The mediators were targeted within weekly *missions* [14] and were assessed using items that have demonstrated acceptable reliability in adolescent populations [25]. However, there are several limitations that should be acknowledged. First, just over half of the adolescents participating in the study provided complete, valid data at each time point, leading to exclusion from the current data analyses. It is possible that the sample size was not sufficient to detect the changes in the assessed variables [24]. Second, due to the sample size, it was not possible to examine mediators separately for males and females, even though there may have been differential effects. Third, the intervention was conducted in low-SES neighborhoods. It must be noted that these results may not apply to the general population in inactive adolescents.

### Conclusions

In conclusion, an intervention effect was only observed for barriers to physical activity, with adolescent’s perceiving more barriers than those in the wait-list control immediately after intervention. However, this did not mediate changes in physical activity and sedentary time at 6-month follow-up. This suggests that the lack of overall intervention effects on physical activity and sedentary time observed in the RAW-PA study could be due to the limited impact of the intervention on the targeted mediators. Future studies should identify intervention strategies that effectively target key mediators to improve physical activity among inactive adolescents. They should also explore additional potential mediators that may explain changes in the use of activity trackers and digital resources over time. Finally, intervention effects according to the level of wearable tracker use or the level of engagement with the intervention should be explored. This information is critical for the design of future successful interventions to increase physical activity in adolescents.

## Abbreviations

LPA: light-intensity physical activity
MVPA: moderate-to-vigorous physical activity
RAW-PA: Raising Awareness of Physical Activity
SEIFA: Socio-Economic Index for Areas
SES: Socioeconomic status
